# The Role of Electrospun Fiber Scaffolds in Stem Cell Therapy for Skin Tissue Regeneration

**DOI:** 10.20900/mo.20190002

**Published:** 2019-02-15

**Authors:** Mulugeta Gizaw, Addison Faglie, Martha Pieper, Sarju Poudel, Shih-Feng Chou

**Affiliations:** Department of Mechanical Engineering, College of Engineering, The University of Texas at Tyler, Tyler, TX 75799, USA

**Keywords:** electrospun fibers, stem cell, tissue engineering, wound healing

## Abstract

Stem cell therapy has emerged as one of the topics in tissue engineering where undifferentiated and multipotent cells are strategically placed/ injected in tissue structure for cell regeneration. Over the years, stem cells have shown promising results in skin repairs for non-healing and/or chronic wounds. The addition of the stem cells around the wound site promotes signaling pathways for growth factors that regulate tissue reconstruction. However, injecting stem cells around the wound site has its drawbacks, including cell death due to lack of microenvironment cues. This particular issue is resolved when biomaterial scaffolds are involved in the cultivation and mechanical support of the stem cells. In this review, we describe the current models of stem cell therapy by injections and those that are done through cell cultures using electrospun fiber scaffolds. Electrospun fibers are considered as an ideal candidate for cell cultures due to their surface properties. Through the control of fiber morphology and fiber structure, cells are able to proliferate and differentiate into keratinocytes for skin tissue regeneration. Furthermore, we provide another perspective of using electrospun fibers and stem cells in a layer-by-layer structure for skin substitutes (dressing). Finally, electrospun fibers have the potential to incorporate bioactive agents to achieve controlled release properties, which is beneficial to the survival of the delivered stem cells or the recruitment of the cells. Overall, our work illustrates that electrospun fibers are ideal for stem cell cultures while serving as cell carriers for wound dressing materials.

## INTRODUCTION

Skin is the outermost tissue that protects us from the outside environment, and it consists of three layers, namely, the inner epidermis, the middle dermis and the innermost hypodermis. Epidermis has no blood vessels and is mainly layers of keratinocytes, Langerhans cells, and melanocytes [1,2]. Keratinocytes represent ectodermal derived cells that make up 80% of the cell population in the epidermis. During injury, stem cells are recruited to the wound site for cell replenishments and repair of the wound by continuously replacing dead cells [1]. Since stem cells are able to self-produce into more stem cells and differentiate into various specialized cells, they have become a promising therapeutic modality to chronic and/or non-healing wounds [3].

In developed countries, the readily available medical care and the abundant social welfare have increased the population of elderly people. According to the United States Census Bureau, population aged 65 and older was 15% of the total population in 2014 and is expected to be 24% of the total population in 2060 [4]. The ability to live longer has generated other health issues, such as diabetics and ulcers (e.g., for those without mobility). Skin regeneration for aged population is slow and represents an important concern with treatment modalities, including patient compliances. As such, chronic wounds are expected to affect 5–7 million elderly patients and cost between US $20-$25 billion annually in the United States alone [5,6]. In underdeveloped countries, where shoes are scarce to children, cutaneous parasitic infections are severe among young populations [7]. Damaged tissues receive little treatment modalities, and patients may need amputation to save their life. Improper healing processes of skin wounds are a significant burden to patient, society, and economy. Non-healing wounds from elderly populations of developed countries as well as young populations of underdeveloped countries have generated increased amount of attentions to find a better mechanistic treatment method involving stems cells on cutaneous regeneration.

Electrospinning was first introduced in the 1930s with electrostatic forces to produce fibers [8]. Currently, electrospinning is utilized to manufacture fiber mats with nonwoven as well as aligned fiber structures, and these fibers, with diameters in the range of several nanometers to micrometers, have attracted applications in wound healing [9,10]. The process of electrospinning, including the preparations of polymer solutions and parameters in electrospinning on fiber size, architecture and mechanical properties has been extensively reviewed previously by others [11–13]. Briefly, a high voltage source is applied to the needle, which is dispensing polymer solutions controlled by the syringe pump. When the electric field overpasses the surface tension of the polymer solution, fibers are drawn and deposited on the collector. Electrospun fibers have demonstrated their potentials in drug delivery and tissue engineering [14,15]. In wound healing, our group has reviewed small molecule drugs as well as biological agents incorporated in and/or immobilized electrospun fibers to provide sustained release purpose so that frequent change of dressing materials can be minimized [16].

**Figure 1.**
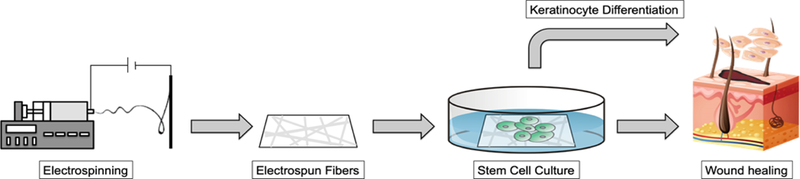
A schematic illustration shows the electrospinning process to produce fiber scaffolds for stem cell cultures. The cultivated cells are then applied either directly or with fiber scaffolds as cell carriers to the wound site to promote wound healing.

The aim of this review is to provide current information on the use of electrospun fiber scaffolds to either incorporate stem cells within fibrous layered structure or use the scaffolds as substrates for stem cell culture to provide healthy cells in skin regeneration. Figure 1 illustrates the theme of our aim in this review, where fiber scaffolds are generated by electrospinning. The resulting fibers, based on fiber porosity, fiber diameter, and fiber alignment, are used for cultivations of stem cells. This technique can be further expanded where a multi-layered fiber scaffolds is in combination with stem cell cultures for dressing materials in tissue regeneration. Our review provides a comprehensive discussion on the current status of electrospun fibers for stem cell cultures in wound healing applications.

## WOUND HEALING

Wound healing is a complex biological process that involves the participation of different immune systems for the regenerations and/or repairs of cells and tissue structures in order to restore its normal biological function [17–19]. Normal and healthy bodies provide regeneration and repair mechanisms on tissue structures when a wound occurs. Specifically, ideal regenerations of tissue structures are involved in the replacements of the damaged cells by keratinocytes. In contrast, normal repair mechanisms typically fill the damaged tissue structures by connective tissues, which later develop into the scar tissues at the wound site.

### Wound Healing Process

**Figure 2.**
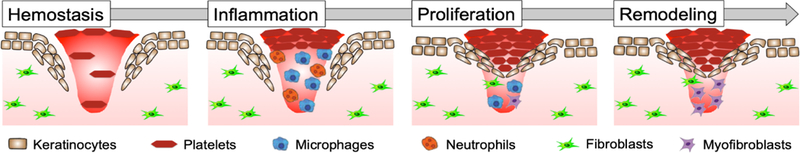
Schematic illustration of a typical wound healing cycle consisting of hemostasis, inflammation, proliferation, and remodeling phases.

A typical wound healing process includes four overlapping and time dependent stages, which are categorized as hemostasis, inflammation, proliferation, and remodeling phases (Figure 2) [20,21]. A normal wound follows these healing steps for tissue regenerations and/or repairs, while a delay or an incomplete stage results in non-healing wounds.

Hemostasis and coagulation is the first stage in wound healing process that takes place immediately at the time of injury and is completed within hours, depending on the wound size and the severity of the wound.

Immediately after skin injury, bleeding takes place with the initiation of hemostasis phase as a result of the presence of high levels of Tissue Factor proteins that activate the clotting cascade to generate fibrin clots [22]. In parallel, platelets are activated after in contact with extracellular matrix (ECM) collagen to form interim platelet plugs for temporary seals of the wound until the fibrin clots are formed to consolidate the wound. Protein and peptide by-products, such as clotting factors, involved in the clotting cascade serve as signals to surrounding cells to recruit immune cells to the wound site forming clots that compose of fibrin molecules, fibronectin, vitronectin, and thrombospondins [19,20,23]. The clots are served as a matrix for immune cells in the subsequent stages of tissue repair and wound healing [21,24].

The second phase in the wound healing process is the inflammatory stage, which is initiated during hemostasis and coagulation phase and can last up to days [25–27]. Inflammations are biological responses to the generations of the by-products from the hemostasis phase, the presence of active platelets and their cytokines, and the release of mediators from in injured cells. Within minutes of injury, neutrophils from microorganisms are released to the wound site. The presences of neutrophils along with monocytes, which later differentiate into macrophages, flight against wound infections. Macrophages facilitate the healing process by ingesting microorganisms, dead neutrophils, fibrin clots, and other cellular debris [19,20]. Macrophages plays an important role in transition of wound site from inflammation to repair by initiating the synthesis of cytokines and growth factors that are crucial in signaling the migration, proliferation, and organization of the newly formed connective tissues and vascular beds. At the end of the inflammatory stage, the wound bed is prepared and cleaned by macrophages suitable for the next healing cycle, proliferation and repair.

The third phase in wound healing cycle is the proliferation stage, and it can last weeks to months to complete. The primary function of this stage includes the formation of newly formed skin (re-epithelialization), the restoration of vascular function to the wound site (neovascularization), and the generation of connective tissue (granulation) [19]. During this stage, fibroblasts and keratinocytes play important roles in modulating the mechanical strength of the wound site. Re-epithelialization of wounds occurs when the surface of the injured skins is covered by keratinocytes. The migration of keratinocytes to the wound site is facilitated by fluid environment and is governed by complex steps regulated by chemotactic gradient produced by different growth factor [19,20]. Re-epithelialization covers the wound with new epidermal cells that minimize the formation of hypertrophic scar tissues. Neovascularization is an important part of the healing process since it restores the vascular network by keeping a steady supply of nutrients. In addition, neovascularization enables formation of ECM with adequate supply of oxygen. Development of granulation tissue is the third and final mechanism in proliferation stage and repair/regeneration of the tissue structures. Most cells found in granulated tissue are fibroblasts, which are dermal cells that produce collagen and ECM. The structure of granulation tissue undergoes constant modifications as it matures. Approximately 3 weeks after injury in a normal healing process, 20% of the final strength of the wound site is restored [28].

Remodeling stage can last from months to years in wound healing [26]. As it is the final phase of wound healing, the remodeling of the tissue structure consists of growth of new epithelium and formation of final scar tissue. The regulations of the epithelium and scar tissue are done by synthesis, deposition, and degradation of the tissues [21]. The wounds contracts and become smaller due to the newly formed collagen matrix that becomes more organized in cell orientation and cross-linked over the phase from a vastly disorganized deposition of preliminary collagen bundles [19,20]. The healed tissue archives 80% of original tensile strength since some cellular components and their structural organizations cannot be fully recovered during healing [24,25].

### Non-Healing Wounds

The most common way to classify wounds is based on the nature of the wound healing process involved (*i.e*., acute and chronic wounds). Specifically, acute wounds are results of mechanical injuries due to external factors such as abrasions and tears to the skin, whereas chronic wounds occur when the normal healing mechanisms in the body are inhibited or when the tissues are constantly exposed to other environmental factors [20,28]. Factors that can impair the healing process resulting in chronic wounds include the presence of foreign bodies, tissue maceration, ischemia, and infections. Other factors that can affect the wound healing process include age, diet, and patients’ medical complications (e.g., diabetes). In addition to these factors, the reduction in tissue growth, the regulation between proteolytic enzymes and their inhibitors, and the presence of senescent cells play important role in chronic wounds development [29]. The most common types of chronic wounds include, venous ulcers, pressure ulcers, and diabetic ulcers [30].

Venous ulcers are the most common lower limb chronic wounds accounting for more than half of lower lib chronic wounds affecting 1%–2% of the adult population. Venous ulcers are typically located between the knees and the ankles [31]. They are caused by venous hypertension and congestion due to venous thrombosis. Due to backpressure in blood vessels, permeability is increased which leads to leakage of macromolecules and red blood cells into the perivascular space where they attract leukocyte infiltration. The diffusion of oxygen and growth factors is impeded by edema and fibrosis. Venous ulcers are usually large and shallow with irregular ill-defined margins [30].

Arterial ulcers are less common than venous ulcers and are caused by insufficient arterial blood flow to the tissue. The insufficient blood flow leads to a decrease of oxygen and nutrients that can be delivered to the wound site and hinders the removal of body fluids from the wound site. Risks that might cause arterial ulcers include smoking, diabetes, hypertension, hypercholesterolemia, and age [32,33].

Pressure ulcers are commonly observed on patients with limited mobility and sensory perceptions. They are caused by a combination of external pressure and shear force. When the external pressure/force exerted on the tissue is higher than normal for a prolonged period of time, the delivery of oxygen and nutrients is hindered resulting in hypoxia and accumulation of waste and free radicals. Skins that are located over bony prominences such as sacrum, hips, and malleoli are usually vulnerable for pressure ulcers [30,31].

Diabetic ulcers are the most common complication for patients with diabetes, and they account for majority of deaths worldwide [34,35]. The poor circulation of the blood prevents the proper delivery of the oxygen and nutrition to the wound site for tissue repairs. In addition, high blood sugar content impedes the healing process of a wound and is likely to cause localized colonization of microorganisms and/or bacteria. Patients with diabetic foot ulcers are vulnerable to reulceration, amputation and death.

### Current Treatments Methods for Non-Healing Wounds

Current management methods for chronic wounds include the use of antimicrobial dressings that aid to the reduction in inflammation and regulation of pathogens [29]. Ideal wound dressing must meet one or several of the following functions: (1) stop bleeding and protect wound from pathogens, (2) restore normal bacterial balance in wound, (3) reduce inflammation due to irregular matrix metalloproteinase, and (4) provide a suitable environment for the control of odor and promotion of autolysis [36].

Based on the types of treatment, wound dressing are categorized in to four groups: passive, interactive, advanced, and bioactive wound dressing. Passive dressings are used to protect mechanical trauma and the entrance of pathogens to the wound. Interactive dressings are made from polymeric films, and they facilitate the flow of moisture and air from the environment while providing a barrier from bacteria or other environmental contaminants. Advanced dressings are able to provide and retain moist environment for the wound and facilitate the healing process. Bioactive dressing works by including drug delivery system and/or biological agents to stimulate cellular responses in the healing process.

Nowadays, regenerative medicine has attracted attentions in wound healing due to the introduction of implantable biomaterials as wound dressings and/or the use of injectable bioengineered stem cells for tissue regeneration [37]. Recent researches have focused on the incorporation of tissue scaffolds and stem cell therapy to promote tissue regenerations [38]. Tissue scaffolds are used for the function to either culture/transport cells as compared to the traditional media method or recruit/signal host cells to repair the damaged tissue. Other methods using regenerative medicines include living cell therapy (Apligraf®) and artificial skins (Integra®) that have been approved by the FDA for treatment of non-healing wounds [39]. To mimic the architecture of the skin tissues, tissue scaffolds are generated in different physical appearances including hydrogels, macro or micro-porous foams, and woven and non-woven medical fabrics. Among them, medical fabrics exhibit a high surface area to volume ratio with a porous structure allowing for the exchange of oxygen and nutrients and the support of cell growth [40,41].

When considering the use of tissue scaffolds for wound healing, several cellular properties, including cell adhesion, migration, proliferation, and differentiation, are important in the promotion of tissue regeneration. Furthermore, scaffold degradation rates in physiological condition need to match the regeneration rate of the new tissues. Lastly, the mechanical properties of the tissue scaffolds should be ideally equal or greater than that of the human skin. The advance in Materials Science and Bioengineering has enabled the development of bioactive dressings using natural/synthetic polymers in the form of fibers as a carrier to deliver drugs and/or biological agents. One of the most popular and promising methods to prepare drug-eluting fibers is by electrospinning. Electrospun fibers exhibit several outstanding properties that make them the preferable candidate for biomedical applications in wound dressing [42]. These advantages include the large surface area to volume ratio [43], the porous nature allowing exchange of oxygen and fluids [16], the ability to immobilize macromolecules to the fiber surface [44], and the independence of mechanical properties to drug loading [45]. Recent studies in regenerative medicines have focused on the use of stem cells integrated electrospun fibers for chronic wound treatment.

## STEM CELLS IN WOUND HEALING

Stem cells have the abilities to produce proregenerative cytokines that promote the healing cascade of a wound [46]. Table 1 lists the use of stem cells by injections around the wound sites of various animal models in wound healing. The regulation and recruitment of the stem cells during wound healing play a significant role in facilitating inflammation and proliferation stages that propagate the release of growth factors, the growth of new blood vessels, and the differentiation of themselves into endothelial cells [47]. While a healthy and normal body exhibits small amounts of blood-borne mesenchymal stem cells (MSCs), others showed that bone marrow-derived stem cells were activated through various signal pathways and eventually reached to the wound site through blood circulation [48,49]. In addition, various sources of stem cells were involved in preclinical or clinical trials for a better understanding of the treatment modalities in ulcers, non-healing wounds, limb ischemia, and burn wounds [50]. In this section, we review the use of mesenchymal stem cells (MSCs) and adipose stem cells (ASCs) in wound healing.

### Mesenchymal Stem Cells

Mesenchymal stem cells (MSCs) are undifferentiated multipotent stem cells, and they are typically isolated from bone marrow, umbilical cord blood, adipose tissues, nerve tissue, amniotic fluid, and dermis. MSCs have shown their effectiveness in treatment of cutaneous wounds through system administration [56] and local injections [46,57]. The presence of MSCs promotes angiogenesis while reducing local inflammation and facilitating extracellular matrix (ECM) formation. In particular, MSCs’ ability to secrete growth factors allows the suppression of local inflammation from the immune system, one of the key mechanisms in non-healing wounds due to a constant apoptosis of healthy cells. These growth factors also stimulate mitosis and cell differentiation with specific phenotypes resulting in the inhibition of the scar tissue (fibroblasts) formation. As such, MSCs’ therapeutic effects in wound healing and tissue regeneration have made them the trophic mediators. In addition to these benefits, MSCs also exhibit antibacterial properties through the similar mechanism in secretion of antimicrobial factors and immune-modulating factors at the wound site to promote immune response to bacteria killing and phagocytosis [58–60]. Furthermore, studies have suggested CCR7, a receptor of SLC/CCL21 expressed by MSCs, was associated with the targeted migrations and homing mechanisms for MSCs to reach the wound site [61].

In parallel with the understanding in MSCs therapeutic effects and migration mechanisms, *in vivo* animal models were used for examinations of various MSCs on the effects of wound closure. For example, adipose tissue derived mesenchymal stem cells (AD-MSCs) showed significant improvements in wound healing of a diabetic rat model [53]. Specifically, AD-MSCs were injected intra-dermally around the skin wound of diabetic rats in comparison with diabetic control groups and non-diabetic control groups. Results suggested a 50% wound closure at 1.5 days, 2.5 days, and 4 days for AD-MSC, non-diabetic, a control, and diabetic control groups, respectively. The corresponding groups achieved full wound closure at around 6 days, 8 days, and 9 days, respectively. Others investigated the use of bone marrow derived stem cells (BMSCs) in combination with thermo-sensitive hydrogels on wound healing of a mice model [54]. Results suggested a 40% wound closure from the control groups, whereas the hydrogel-BMSCs achieved 60% of wound closure after 3 days. At 7 days, the control groups reached 80% wound closure and the hydrogel-BMSCs showed a full wound closure (100%) with histological results supporting the full re-epithelialization of the skin tissue. In addition, studies showed that MSCs promoted proliferation phase and inflammatory phase in wound healing resulting in a faster healing rate [62]. Specifically, caprine amniotic fluid (cAF) and bone marrow cells (cBM) derived MSCs were injected subcutaneously around the wound edge of a rabbit model. Results suggested a 20% reduction of the wound from cAF-MSC and cBM-MSC groups as compared to the 17% closure from the control groups. Furthermore, cAF-MSC and cBM-MSC groups achieved 85% and 75% of wound closure at 21 days, respectively, as compared to the 65% closure from the control groups. Others compared the effectiveness of wound healing in diabetic mouse models by injecting BMSCs and fibroblasts to the wound sites [63]. Results suggested an 85% of wound closure from BMSC groups and a 65% wound closure from fibroblast groups after 28 days. In another study, burn-derived mesenchymal stem cells (BD-MSCs), obtained from full-thickness burned skin (*i.e.,* third-degree burn), were incorporated into MatrigelTM for investigation of wound closure rate in mouse models [64]. Results suggested that mice received BD-MSCs healed faster than the control groups, and histological examinations showed that BD-MSCs administered mice had a smaller wound size and a thinner keratinocyte layer than the control groups. These examples suggested the effectiveness in treatment of wound healing using stem cell therapy.

### Adipose Stem Cells

Adipose stem cells (ASC) are also undifferentiated multipotent stem cells that can be extracted from adipose tissues. It has been shown that stem cells obtained from adipose tissues had a 40-fold yield than those obtained from the bone marrows [65]. Furthermore, studies showed that the ASC culture media exhibited various concentrations of transforming growth factor beta, vascular endothelial growth factor, keratinocyte growth factor, fibroblast growth factor 2, platelet-derived growth factor, hepatocyte growth factor, fibronectin, and collagen I [66]. With the ability to secrete wound healing related growth factors, ASCs are considered a prime candidate for cell therapy in wound healing.

The presence of ASCs in the culture media or a wound bed upregulates the biological activities and crosstalks between cells by secreting wound healing factors (e.g., insulin-like growth factor, hepatocyte growth factor, and vascular endothelial growth factor) to stimulate recruitment, migration, and proliferation of endogenous cells in the wound environment. For example, *in vitro* cultures of human dermal fibroblasts (HDF) and ASCs showed a significant increase in HDF population (67%) as compared to the control groups (30%) after 2 days [67]. In addition, type I collagen secretion from HDF suggested a dose-dependent relationship with ASC concentrations and achieved a 2-fold increase as compared to the control groups. Furthermore, treatment with ASCs showed a faster wound healing after 7 day on mice with a wound reduction area of 34% as compared to the control groups. Others demonstrated that the effects of hypoxia (2% O2) promoted the proliferation of ASCs after 72 h in serum-free culture media, where a significantly higher type I collagen secretion was found using the ASC-conditioned media for HDF cultures [68]. Furthermore, wound area decreased by 27% on mice models after receiving ASC-conditioned media as compared to the control groups after 4 days. In another study, ASCs were injected around the wounds that were created on the back of Yorkshire pigs [51]. Results showed that not only ASCs were compatible with the wound environment (e.g., no noticeable immune responses) but also high dosage of ACSs achieved faster wound closure rates (e.g., 48.0% and 65.5%) as compared to the controls (e.g., 37.9% and 48.9%) at 10 and 14 days, respectively. In particular, ASCs were responsible for secreting vascular endothelial growth factor for the remodeling of collagen matrix resulting in the improvement of scar quality. Similarly, others incorporated over-expressing vascular endothelial growth factor in nanoparticles and delivered with ASCs to the wound site using mouse models. Results suggested that ASC groups achieved a complete wound healing in 8 days as compared to 12 days from the control groups [52]. These examples demonstrate the roles of ASCs and their abilities to promote wound healing.

### Role of Fiber Scaffolds on Stem Cells in Tissue Regeneration

The primary objectives of wound management are healing and pain control. Innovations in wound dressings have been developed to facilitate wound care. An array of pharmaceutical therapies on wound health has been proposed in recent years, including the promising gene delivery and stem cell treatments [69]. However, current treatment modalities involved in wound healing using stem cell therapy often require the injection of cells around the wound bed. This approach depends critically on cell survival after injection where the microenvironment cues play an important role on the promotion of cell proliferation and differentiation. Other challenges in gene delivery and stem cell therapy include achieving a better selection of target cells, elaboration on personalized therapeutic methods, and diagnosis of factors affecting the wound healing process [69]. In this regards, tissue scaffolds provide benefits in controllable outcomes for gene delivery and stem cell therapy where tissue regeneration depends on a suitable tissue scaffold, delivery of the biological agents, and hosting of cells for tissue reconstruction (Table 2). Currently research suggests that the reproduction of 3D tissue scaffolds that mimic ECM is ideal for cell proliferation and growth [70]. As a result, cellular delivery in a bioactive fibrous scaffold has emerged as a promising therapeutic method [71].

**Figure 3.**
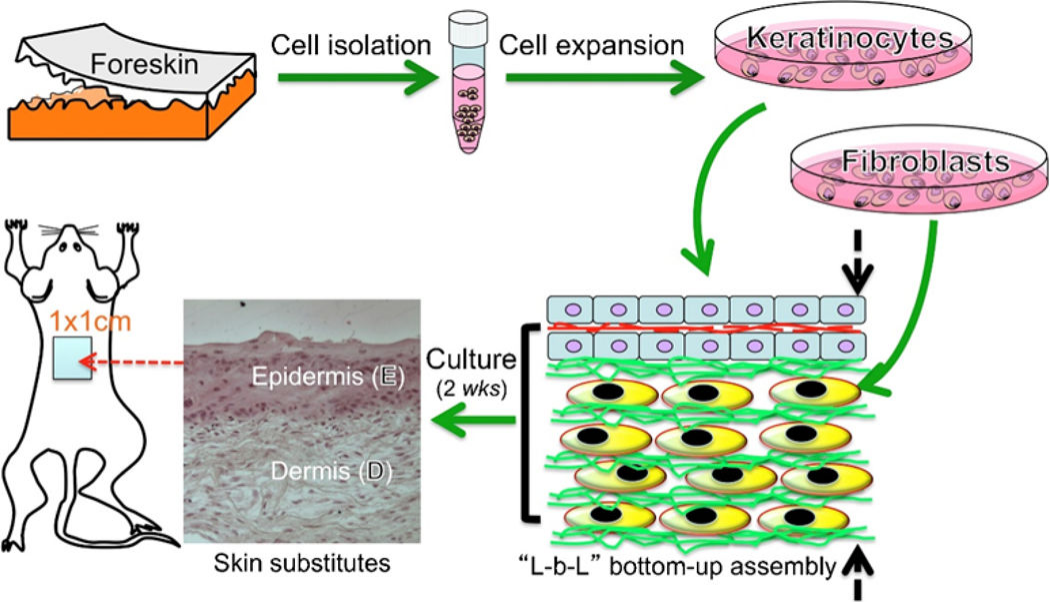
Generation of a full-thickness skin substitutes as wound dressings developed from layer-by-layer nanofiber/cell assembly. Reprinted from [72], Copyright © 2015, with permission from Elsevier.

The use of fiber scaffolds in culture and delivery of cells are promising for wound healing. In a study, a skin substitute was developed for topical application on burn wounds using mice models (Figure 3) [72]. The skin substitute was made from electrospun polycaprolactone/collagen fibers as a composite material to incorporate layers of keratinocytes and fibroblasts at top and bottom of the 3D construct, respectively. Results from the animal study showed that blank scaffolds received the lowest score in integration of the wound whereas the layered 3D constructs integrated with the wound bed and became unrecognized after 21 days. In particular, at 21 days, wound treated with layered 3D constructs had 7% remaining in re-epithelialization with a 45% wound contraction as compared to only 21% re-epithelialization with 56% wound contraction on the blank fiber groups. The study suggested that the 3D layered tissue constructs was comparable to skin substitutes, which led to regeneration of full tissue and healing of a burn wound on a mice model. Others demonstrated on the use of 3D printed chitosan scaffolds as tissue substitutes to culture fibroblasts and keratinocytes for 20 to 35 days [70]. Result from diabetic-induced rat models after 14 days suggested that the implanted scaffolds on wounds accelerate healing as compared to the control groups. This study indicated that 3D scaffolds provide a better biometric milieu than the 2D conventional culture. In another study, endothelial progenitor cells (EPCs) were cultured for 48 h on fibrous composite scaffolds consisted of polycaprolactone/collagen fibers and bioactive glass nanoparticles [71]. Results from wound healing using rat models showed formation of the blood vessels and an upregulation on Hif-1α, VEGF, and SDF-1 α expression, indicating the promotion in angiogenesis. 100% wound closure was achieved using the fibrous composite scaffolds at 21 days as compared to 80% of the wound closure from the control groups.

Incorporation of MSCs with porous scaffolds has the potential in wound healing as compared to direction injections of the cell cultures. For example, MSCs were loaded into rosuvastatin calcium incorporated porous scaffolds, made from lyophilization of crosslinked chitosan hydrochloride with collagen, β-glycerolphosphate, and carboxymethyl cellulose, to investigate the healing properties of surgical induced wounds using rat models [75]. Results suggested a sustained release of rosuvastatin calcium over 60 h with approximately a 2-fold increase in proliferation of human dermal fibroblasts after 72 h. Furthermore, the MSC-loaded and drug-incorporated scaffolds showed a 40% reduction in wound size as compared to the 90% wound closure from control groups at 7 days. 100% reduction in wound size was achieved from the scaffolds at 7 days, whereas the control groups reached 65% wound closure. Others seeded MSCs on poly(D,L-lactic acid) (with and/or without the incorporation of a biomass, *Spirulina*) fibrous scaffolds to improve wound healing using mice models [74]. Results showed that biomass-incorporated fiber scaffolds adhered better on the wound than the counterpart (fibers without biomass). After 7 days, the MSC-seeded and biomass-incorporated fiber scaffold showed better macroscopic tissue regeneration. Similarly, studies showed that biomimetic fiber scaffolds with attachment of bone marrow derived MSCs accelerated the healing process on an acute full-thickness skin wound using rat models [73]. Results showed that the wounds were fully epithelialized after 10 days for the fiber scaffold seeded with high density of the stem cells as compared to 14–15 days of healing for the control groups.

## ELECTROSPUN FIBER SCAFFOLDS MEDIATED STEM CELL THERAPY

Technological improvements and understandings in electrospinning have made electrospun fibers an attractive candidate for biomedical applications. Electrospun fibers have the potentials and capabilities in promoting wound healing, drug delivery, and tissue engineering. In addition, the possible materials that can be electrospun into fibers are almost endless including both natural and synthetic polymers as well as blends to give a large degree of variation in the characteristics of the fibers [16]. Table 3 summarizes the use of various electrospun fibers on the *in vitro* culture of cells for skin tissue regeneration. Furthermore, electrospinning process is relatively low cost where the non-woven fibers mates are highly porous and lightweight due to the large surface area to volume ratio. The physical properties of fibers, including fiber morphologies and fiber diameters, are also relatively easy to control resulting in predictable fiber physicochemical characteristics for biomedical functions. However, the only main drawback of electrospun nanofibers in stem cell culture involves in the tightly stacked fibers, which may result in the restriction of cells and nutrients infiltration through the fibers. To work around this particular issue, studies suggested that incorporating Eudragit in fibers made from blends of chitosan, gelatin, and polyethylene oxide followed by the removal of the Eudragit enhanced the permeability of fiber membranes [76].

### Fiber Porosity, Diameter, Alignment

**Figure 4.**
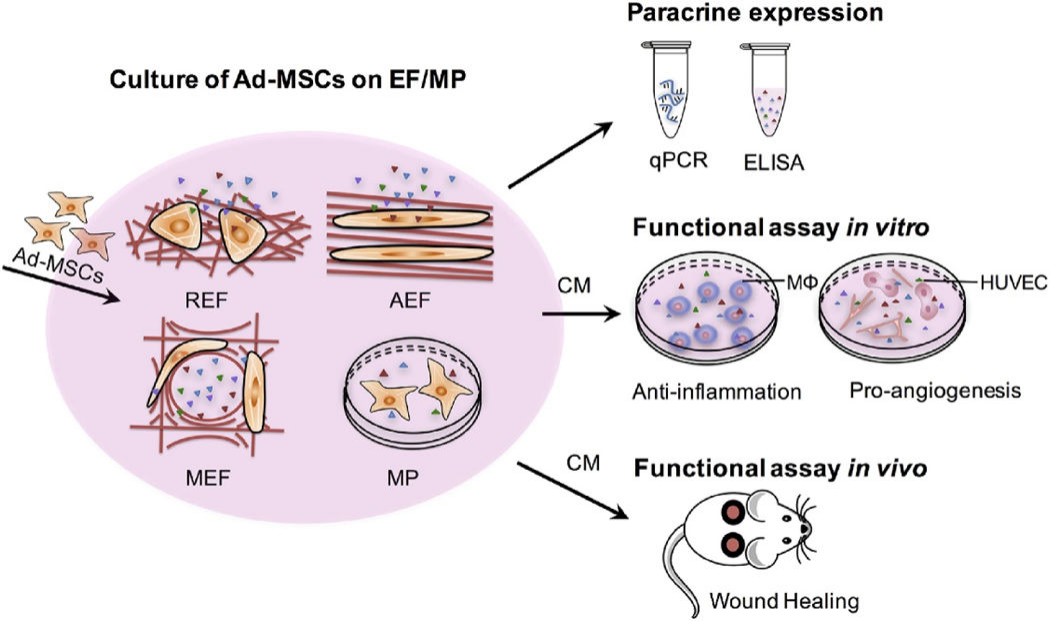
Study design on the effects of fiber morphology and fiber orientation on the paracrine secretion and function of AD-MSCs. Electrospun fiber scaffolds for cell culture included random (REF), aligned (AEF) and a mesh organization (MEF) for comparison with cultures on polystyrene microplate (MP) [83]. Reused from [83], an open access article distributed under the CC BY-NC-ND license (http://creativecommons.org/licenses/by-nc-nd/4.0/).

It has been shown that fiber structures, including porosity of the fiber mats, fiber diameter, and fiber alignment, play a significant role in the culture of stem cells (Figure 4) [83]. For example, a fibrous substrate with a high porosity as well as a high amount of interconnected pores allows a three-dimensional arrangement of stem cells. The pores provide passage for molecules to communicate through the extracellular space as well as to promote the adhering of the cells to the matrix structure. The pores are also essential for cell survival due to the need for the exchange of nutrients [74]. When using fiber membranes for dressing, fiber porosity is essential for cell attachment and growth, drainage of the wound exudates, and permeation of atmospheric oxygen to the wound [84]. Studies showed that porous fiber mats were ideal for cell infiltration and exchange of oxygen and nutrients, and hence, increasing cell proliferation [85]. Others showed that fiber scaffolds provided the mechanical support of newly formed tissue by interlocking the fibers with the surrounding tissue [86]. Electrospun fibers scaffolds used for tissue engineering usually have fiber porosity higher than 80% [87]. In another study, the rate of MSC proliferation depended on the polyethylene terephthalate fiber porosity with higher porosity increased cell proliferation [88]. Similarly, porosity of the poly(L-lactide) and blends of poly(L-lactide) and poly(glycolic acid) fibers affected the differentiation of neural crest cell-like synovial stem cells with higher porosity fibers increased the expression of chondrogenic and osteogenic genes while suppressing smooth muscles and adipogenic genes [89].

Fiber diameter is governed by the whipping elongation as a result of the electric field and the viscoelastic forces due to solvent evaporation. Adjusting of fiber diameter in electrospinning is achieved by altering solution viscosity, specific electric field, and the evaporation rate of the solvent [90]. It has been demonstrated that decreasing fiber diameter resulted in a greater rate of cell spreading and proliferation with a lower rate of cell aggregation [91]. Results showed that the proliferation and differentiation of cultured rat hippocampus-derived adult NSCs (rNSCs) had strong influences on the diameter of the laminin-coated electrospun polyethersulfone (PES) fiber meshes. In particular, a 40% increase in oligodendrocyte differentiation and a 20% increase in neuronal differentiation were found using fibers with average fiber diameter of 283 nm and 749 nm, respectively, in comparison to the controls of tissue culture polystyrene substrates. Others have demonstrated that large surface area (*i.e.,* small fiber diameter) of the blend fibers from mucilage and poly(vinyl alcohol) allowed proper adhering and proliferation of the L929 fibroblasts cells [92]. In another study, randomly oriented poly(vinyl alcohol) fibers with average diameters ranged from 70 nm to 1120 nm affects cell responses [93]. Furthermore, the studies showed that the thickness of a fiber mat affected the size, morphology and actine organization of keratinocytes much more than fibroblasts.

Fiber orientation has also been shown to impact cell responses. Fibers with an aligned orientation appear to facilitate greater cell elongation as well as cell growth compared to randomly aligned fibers [94]. For example, aligned electrospun fibers have been demonstrated to guide the highly organized extracellular matrix when being deposited onto a surface [95]. Results showed the ability to direct MSCs using aligned electrospun fibers towards either a ligamentogenic, chondrogenic or fibrochondrogenic phenotype. This suggests a possible ability to control MSCs differentiation through the adjustment and application of fiber alignments. More evidence of the importance of fiber alignment can be seen from a study that randomly oriented or unstructured fiber surfaces resulted in a material similar to the structure of pathological tendon [96]. Fiber scaffolds with a disorganized alignment showed a negatively effect on early cell attachment as well as gene expression. Others demonstrated that electrospun fibers with controlled fiber alignment at various degrees of alignment (e.g., non-aligned, moderately-aligned, and highly-aligned) resulted in directional growth of human astrocytoma cells from the highly-aligned and moderately-aligned fibers [97]. In addition, the aspect ratio of cells was found to increase with an increase in degree of alignment in fiber scaffolds. The highly-aligned fiber scaffolds were concluded to have the most potential in neural tissue engineering.

### Fiber Degradation and Cytotoxicity

The degradation rate of fiber scaffolds significantly affects tissue regeneration. Ideal scaffolds are expected to degrade at a rate that matches tissue regeneration rate, and polymer degradation is a determinant factor in tissue engineering [98]. In addition, the by-products from polymer degradation need to be nontoxic and metabolizable for cells and tissues. Biocompatibility between fiber scaffolds and cells is regulated by surface interactions, which affect the regulation of cell activities, including cell migration and adhesion. Therefore, proper selections of biomaterials in fiber scaffolds are crucial as it occupies a key role in biocompatibility and cell growth.

Low toxicity, biocompatibility, and biodegradability are all essential factors to consider when selecting the proper materials to utilize. There are many acceptable natural polymers that fit the desired characteristics in cell compatibility, including polysaccharides, spirulina, and alginate. Polysaccharides have an excellent biocompatibility and reasonable biodegradation rates while they also possess a relatively low cost. Plant mucilage is also a good material for use as a biomaterial scaffold as it promotes cell growth as well as having antioxidant, anti-inflammatory, and antibacterial properties [92]. Spirulina, on the other hand, is a material best used in combination with another polymer, preferably one that is biocompatible and biodegradable. The properties of spirulina have been shown to improve cell viability and cell adhesion of stem cells as compared to the properties of the polymer without the addition of spirulina [74]. Alginate is biocompatible, biodegradable, and has low cytotoxicity. Studies showed that there was no significant difference in cytotoxicity levels between alginate/gelatin hydrogel fibers and alginate fibers [99]. Lastly, a very popular and widely used biocompatible polymer is poly(vinyl alcohol) (PVA), and it is particularly useful as an additive or used in a blended fiber. In a study, PVA was used as an aiding agent in order to promote biocompatibility for cell growth in fiber scaffolds made from mucilage [92]. The results of the study showed that the addition of PVA promoted the formation of fibers in electrospinning and improved biocompatibility.

### Proliferation

Cell proliferation is important in stem cell cultivation as it involves the amount and rate of new cells replicated from the initial cells. Since fiber topography, especially fiber diameter, has a great influence in cell proliferation, it is important to select the proper polymers for electrospinning. For example, it has been shown that blend fibers from polycaprolactone and collagen promoted the proliferation of both human dermal fibroblasts and human epidermal keratinocytes [72]. After analyzing the skin substitutes for total DNA, collagen, and glycosaminoglycan, results suggested that the cells in the assembled constructs continuously proliferated and deposited new ECM with an increase in glycosaminoglycan indicating the promotion of cell proliferation. It has been shown that hydrogels lacking macro-porosity hinder proliferation and cell motility. For example, studies showed that cells seeded on alginate fibers displayed a 8-fold proliferation over 5 weeks of culture on MSCs [99]. In another study, improvement on cell proliferation was found on using a fibrous scaffold without altering the phenotype [100]. Results suggested that using fiber scaffolds allowed the modifications of cells to recover, grow and proliferate. This showed that using electrospun fiber scaffolds as substrates for growth of genetically modified cells aided in the probability of successful gene editing.

### Differentiation

An important topic in stem cell culture for wound healing is the differentiation of the cells. Stem cell differentiation involves the ability or process of an unspecialized cell changing into a specialized cell, like a nerve, skin, muscle, brain and bone cells to name a few. There are many promising applications involving differentiation including a tissue source for transplantation therapy, creation of insulin-secreting pancreas cells to potentially treat diabetes, utilizing specialized cells to study human diseases in the laboratory, and of course wound healing using nanofiber delivery systems [101]. For example, sponges made from electrospun fibers using tilapia skin collagen were developed for wound dressing applications [102]. Results showed that the fibers exhibited good swelling property, thermal stability, and good bioactivity with the ability to rapidly accelerate wound healing of rat models. Most importantly, the fibers demonstrated the ability to promote the proliferation of human keratinocytes and stimulate epidermal differentiation.

MSCs have been shown to differentiate into adipocytes, chondrocytes, myocytes, and osteocytes. They have also been demonstrated to repair tissues by directly differentiating toward mesenchymal lineages [74]. In another study, biodegradable fiber scaffolds were prepared by electrospinning poly(lactic-*co-*glycolic acid) (PLGA) and incorporated into hybrid scaffolds of hydroxyapatite-PLGA/gelatin and PLGA/gelatin [103]. MSCs were isolated from adipose tissues and cultured onto the electrospun scaffolds, and results showed that MSCs on hydroxyapatite- PLGA/gelatin exhibited greater osteogenic differentiation than PLGA/gelatin. It has been demonstrated that MSCs are key players in tissue regeneration due to their capability to differentiate into multilineages. MSCs have a poor cell engraftment resulting in diminished survivability drawing into question the effectiveness of the therapy. However, studies suggested that alginate fibers were able to act as stem cell carriers to support stem cell growth and survival [99].

## FUNCTIONAL FIBERS FOR STEM CELL THERAPY

Stem cells are undifferentiated cells with undetermined functions, and they remain in such state before receiving signals and/or stimulations to become specialized toward a particular cell type [104]. As signals enter the stem cells and differentiations begin, genes that are required for a specialized function remain open and active [105–107]. For example, human embryonic stem cells (hESCs) requires specific signals to initiate the differentiation in a controlled manner, to regulate and/or shut down their growth and progeny once they have been transferred to the recipient, and to circumvent the recognition of non-autologous hESC-derived cells as foreign [108]. The gene therapy technologypossesses a significant potential to deliver biological agents as the specific signals for controlled stem cell differentiation before its transplantation to the recipient. The gene directs the production and use of different proteins and peptides to provide the essential signals for differentiating into a target cell type [109].

### Fibers with DNA

**Figure 5.**
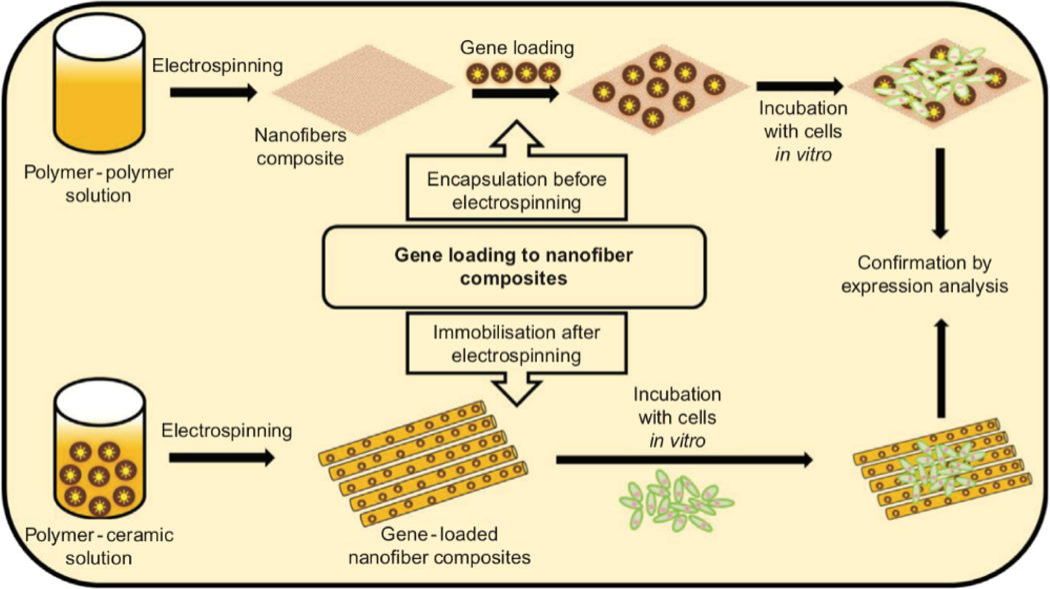
Schematic illustration of common methods in gene incorporated fibers for gene delivery. Reprinted from [112], Copyright © 2017, with permission from Elsevier.

Introduction of DNA into the stem cells can signal the differentiation of cells into cells of interests (Figure 5) [110–112]. The insertion of DNA strand (gene) into a cell can be achieved by viral vectors and non-viral vectors [113]. Electrospun fibers are versatile carriers for biological agents in gene delivery to promote stem cell therapy. Several studies have focused on the release of bioactive molecules for regeneration of tissue. One of the methods widely implemented is fabrication of fibers containing bioactive agents (growth factors, plasmid DNA, viruses for gen delivery) [114]. The incorporation of DNA into fibers is achieved by using non-viral gene vectors such as plasmid DNA or DNA/polyplexes and viral vectors.

Virus has capacity to replace the host’s cell genome with its genome and cause the expression of viral genome in the cells. Several viruses like retrovirus, adenovirus, pox virus, and lentivirus have been used as the medium of gene therapy [115]. Transfection with viral vector requires each complementary DNA (cDNA) or therapeutic gene to be cloned with specific vectors [116]. The therapeutic genes are first inserted into thenon-essential site of viral genome in virus, which transduces the recombinant gene into the stem cells. This method of gene delivery has greater challenges in loading the therapeutic gene in virus, which possess a high risk as the recombinant DNA can possibly revert back to viral DNA leading to impaired immune system in the recipient. Therefore, the alternative method to gene transportation is the use of non-viral vector.

**Figure 6.**
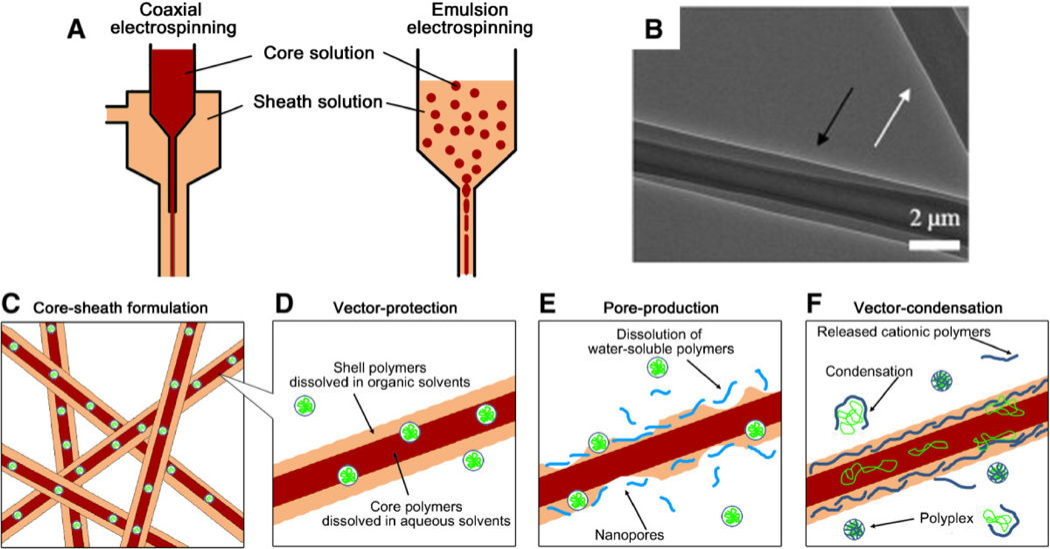
Core-shell electrospun fibers for controlled gene delivery [117]. (**A**) Two representative methods to form core-shell electrospun fibers: coaxial (left) and emulsion (right). (**B**) Transmission electron microscopy (TEM) image of an individual core-shell fiber fabricated using coaxial electrospinning. Reprinted from [123], Copyright © 2009, with permission from Elsevier. (**C**) A scheme depicting gene vector encapsulation within the core layer for controlled release. The core-shell fiber formulations contribute (**D**) to preventing the direct contact of gene vectors in the core layer with organic solvents in the shell layer, (**E**) to regulating delivery modes by producing porous shell layers, and (**F**) to enhancing delivery efficiencies by modifying the shell layers with polycationic polymers. Reused from [117], an open access article distributed under the Creative Commons Attribution License (http://creativecommons.org/licenses/by/4.0)).

Non-viral vectors are used for their ease of production while viral vectors are used for their capacity to increase gene delivery efficiency or extend the duration of gene expression (Figure 6) [117]. Poly(ethylenimine) (PEI) polymer has been used for transfection due its high affinity with DNA to form the complex, osmotic swelling and rupture of endosomes releasing DNA into nucleus, efficient protection from lysosomes, and formation of nanosized complex [110,118]. In a study, fiber scaffolds electrospun from PEI and hyaluronic acid (HA) blends were used for gene delivery [119]. The fibers consisted of core-shell structure, with the outer part containing PEI-HA and the inner part containing DNA-enhanced green fluorescent protein (EGFP). Results showed that fibroblast-like cells seeded directly onto fiber scaffolds upregulated EGFP expression over 60 days as compared to fibers containing plasmid DNA alone. Another widely used polymer, namely polyplex, is able to carry a specific ligand that is recognized by cell membrane receptors [120]. The ligand present in the polyplex attaches it to the cell membrane, and polyplex is taken by the host stem cells during the endocytosis. Due to the internalization of the plasmid DNA inside the positive complex, therapeutic genes remain protected from lysosomes present in cytoplasm. The polyplex when enters the endoplasm releases the DNA by dissolving the polyplex membrane. The released genome in the endoplasm of nucleus was translocated with the stem cell’s unorganized DNA. The stem cell, therefore, identifies itself as a unique differentiated cell and performs the desired function in the recipient body [121]. Furthermore, studies demonstrated the transfer of DNA using PEI polymer in both *in vivo* and *in vitro* cultures. In the *in vitro* cultures, solutions of plasmid DNA (rhodamine-conjugated oligonucleotide) and PEI solution in predetermined ratios were added to the chicken embryonic neurons cells for 24 h. Results showed that luciferase expression was highest in cells transfected with complexes containing about 10 PEI nitrogen per DNA phosphate. In the *in vivo* transfection of intracerebral gene to mice brain, results were supportive to the *in vitro* tests when using PEI-DNA polyplex [118]. In another study, the surface of the electrospun polycaprolactone fibers were immobilized with runt-related transcription factor 2 (RUNX2) using a liposome structure [122]. *In vitro* culture of human bone-marrow- derived MSCs (hBMSCs) using the RUNX2-immobilized fiber scaffolds suggested an enhanced level of metabolic activity and total protein synthesis. Overall, gene delivery using fiber scaffolds shows great potential in activation and stimulation of stem cell differentiation to promote wound healing.

### Fibers with Proteins/Peptides

**Figure 7.**
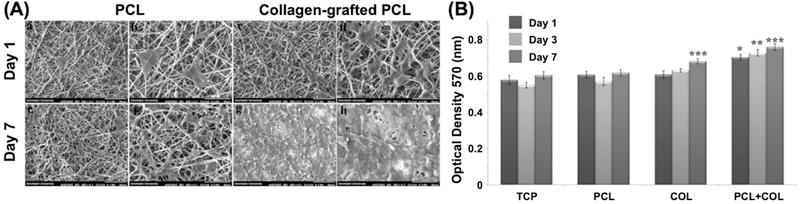
(**A**) SEM image of hEnSCs attachment on nanofiber mats; showing hEnSCs attachment on day 1 and 7 of cell seeding on bare PCL (a-d) and PCL/collagen (e-h). hEnSCs fully covered PCL/collagen construct at day 7, compared to PCL mat alone. (**B**) Cell viability measurements of hEnSCs on PCL, collagen, and PCL/collagen nanofiber scaffolds. Cell proliferation on collagen-coated PCL nanofiber shows significant improvement as compared to bare PCL after 3 and 7 days seeding of hEnSCs. Reprinted from [124], Copyright © 2018, with permission from Wiley.

Stem cell therapy possesses a promising solution to overcome many challenges in acute and chronic wound. Depending on the types of wounds, healing typically requires a large number of homologous stem cells that are compatible with the recipient body. To obtain such cells, the substrates used in the cultures are favorable when including complex mixtures of ECM components, proteins, and growth factors in the soluble media. The protein present on the substrate helps to increase the cell attachment and proliferation on the substrate. While several biomimetic skin substitutes have been studied, one of the approaches is to electrospun fibers containing bioactive proteins. For example, collagen is a natural protein that makes major part of ECM while its structural organization significantly affects the mechanical properties of skin. In a study, 3D polycaprolactone/collagen scaffolds were electrospun for skin reconstruction using human endometrial stem cells (hEnSCs) seeded on the scaffold (Figure 7) [124]. Results showed a higher wettability with desired mechanical and structural characteristics for polycaprolactone/ collagen fibers as compared to the pure polycaprolactone and collagen fibers alone. Attachment of hEnSCs on the polycaprolactone/collagen fibers was higher than the pure polycaprolactone or collagen fibers.

Growth factors are incorporated within the fibers using core-shell configurations to improve skin regeneration (Table 4). In core-shell electrospinning, fibers are fabricated using a specialized spinning nozzle containing a core compartment that sprays the bioactive agents. Core-shell setup is preferred for stem cell differentiation since it provides a sustained release of the bioactive agents. For example, basic fibroblast growth factor (bFGF) was loaded within the core of poly(lactic-*co*-glycolic) acid fibers using coaxial electrospinning as well as homogeneously dispersed within the fiber using regular electrospinning method [125]. bFGF plays an important role in MSCs’ proliferation and differentiation hence promoting tissue repair. Results showed that both groups achieved a sustained release for 1 week, with the coaxial groups sustained the release of bFGF for up to 14 days while the uniaxial fibers released all bFGF within 7 days. While both coaxial fibers and uniaxial fibers supported high levels of attachment and proliferation from bone marrow stem cells, uniaxial fibers promoted collagen production and upregulated gene expression. Although the coaxial fibers sustained the release of bFGF, the relatively low cell activities may be contributing to fiber properties, such as surface topology, surface chemistry, and surface energy. In another study, coaxial electrospinning was used to encapsulate photosensitive polymer poly(3-hexylthiophene) (P3HT) and epidermal growth factor (EGF) in a core-shell structured gelatin/ poly(L-lactic acid)-*co*-poly-(ε-caprolactone) fibers as novel skin graft [126,127]. *In vitro* studies showed complete wound closure within 9 days under light stimulation. Furthermore, ASCs were found to differentiate into keratinocytes under light stimulation using the core-shell fibers. In general, core-shell fibers can be useful in strategically placing the bioactive agents within the fibers to achieve sustained release.

## FIBER COMPOSITES AS DRESSINGS

Recent research has focused on the use of composite biomaterials for stem cell therapy in wound healing. The use of composite biomaterials is advantageous in a way that the lack of desired property from one material is compensated by the others.

### Hydrogel-Fiber Composites

Hydrogels are the most commonly used scaffolds for tissue engineering. However, hydrogels are characterized by low mechanical properties, rapid release of bioactive agents due to high water content, and inability to guide cell proliferation and differentiation that limit their applications in tissue engineering. Hence, the incorporations of fibers in hydrogels are a potential solution to these issues. In a study, electrospun polycaprolactone fibers were incorporated into gelatin hydrogels, and the compressive Young’s modulus increased from 3.3 kPa to 20.3 kPa [129]. In addition, results from proliferation assays and immunostaining analyses suggested that the fibers reinforced hydrogels were compatible with bone marrow MSCs with an enhancement in cell proliferation. The findings of this work demonstrated an improvement in cell delivery for tissue regeneration when using fiber/hydrogel composites.

Hydrogels by nature are hydrophilic, and the incorporation of hydrophobic electrospun fibers allows the adjustment of hydrophilicity/ hydrophobicity of the culture scaffolds. For example, polycaprolactone is a widely applicable hydrophobic synthetic biomaterial for electrospinning of fibers. The incorporation of hydrophobic polycaprolactone fibers in hydrophilic hydrogels, made from methacrylated hyaluronic acid and methacrylated gelatin, was demonstrated in a study [130]. The polycaprolactone fibers were treated with poly(glycerol sebacate) to adjust the surface hydrophobicity of the fibers when incorporating into hydrogels. Results showed that fiber/hydrogel composites exhibited a significant higher level of metabolic activities of sheep mitral valvular interstitial cells after 21 days of culture as compared to the pure hydrogel and fibers alone.

Another limitation of hydrogels in biomedical application is their poor influence on guiding cell growth. Incorporation of electrospun fibers has been shown to improve controlled cell growth direction by serving as guidance. In a study, laminin-coated polycaprolactone fibers were incorporated with hyaluronic acid hydrogels to provide culture support for neuronal cells [131]. Results showed that fiber/hydrogel composites improved the alignment of neurites and their distance of extension as compared to plain polycaprolactone fibers as well as plain hyaluronic acid hydrogels.

**Figure 8.**
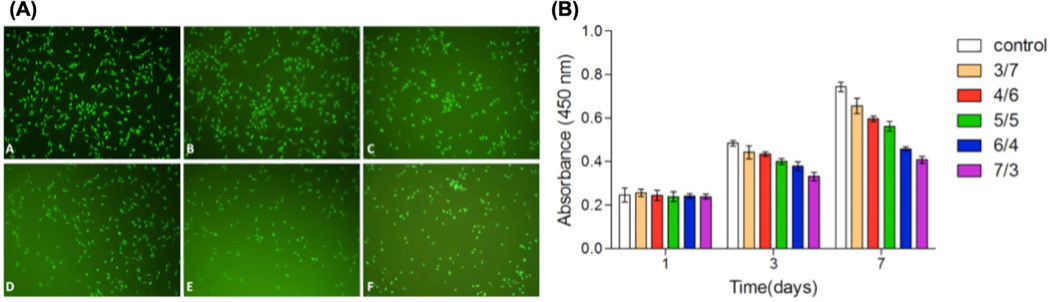
(**a**) Proliferation of cells cultured on dextran/gelatin hydrogels in the CCK-8 assay: (A) tissue culture plate, (B) 3/7, (C) 4/6, (D) 5/5, (E) 6/4, and (F) 7/3 of dextran/gelatin. (**b**) Quantifications of cell proliferations for 1, 3, and 7 days on the corresponding substrates. Reused from [132], an open access article distributed under the Creative Commons Attribution License (http://creativecommons.org/licenses/by/4.0).

Others demonstrated the use of bilayer biomaterials consisting of electrospun poly(e-caprolactone-co-lactide)/poloxamer (PLCL/Poloxamer) fibers and dextran/gelatin hydrogels (Figure 8) [132]. PLCL/poloxamer (9/1) fibers showed the highest mechanical properties at various PLCL/ poloxamer ratios. In addition, the bilayer structure had the advantage of providing mechanical strength from the fiber layer while hydrogel layer was suitable for cell proliferation and generation of ECM. *In vitro* degradation of hydrogel with different ratios of dextran/gelatin hydrogels showed that dextran/gelatin at 3/7 ratio received the highest degradation rate with 100% degradation in 7 days. The 4/6 and 5/5 groups maintained a controlled degradation, while the 6/4 and 7/3 groups showed slow degradation rate with only 50% of degradation after 3 weeks. The mechanical strength of the fibers and the controllable degradation rates from hydrogels allow the engineering of the composite tissue constructs.

### Nanoparticle-Fiber Composites

**Figure 9.**
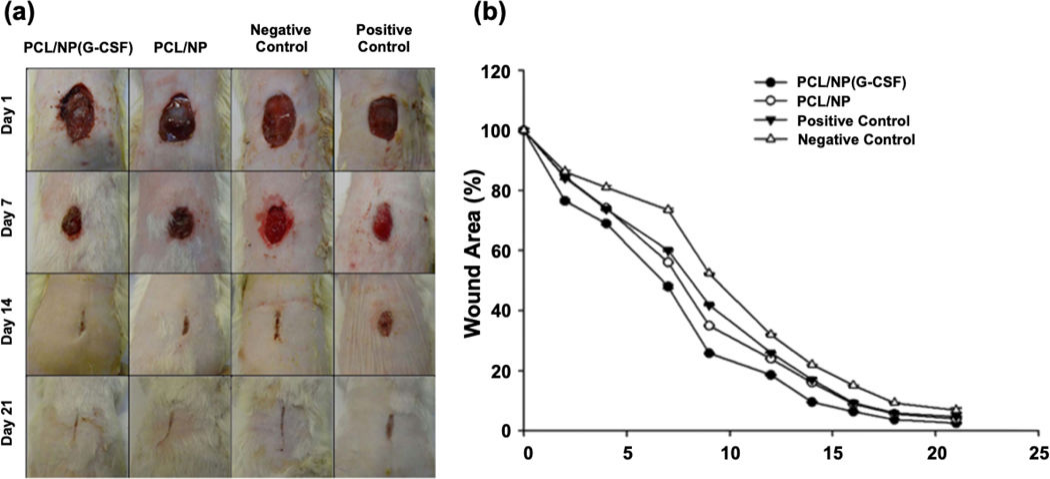
(**a**) Morphological evaluation of wounds healing: on days 1, 7, 14, and 21 days using PCL/NP(G-CSF), PCL/NP, positive control and negative control. (**b**) Wound area percentage for *in vivo* wound healing using PCL/NP(G-CSF), PCL/NP, positive and negative control. Reprinted from [134],Copyright © 2017, with permission from Wiley.

Preparation of nanoparticle-embedded fiber dressings has been widely studied for their applications in wound healing and tissue engineering. The composite materials have high mechanical integrity, cell support and guidance, and multiple release kinetics of encapsulated substances [133]. Due to their small size, nanoparticles have potential to be used as carriers for biological agents in hard to reach areas. However, nanoparticles require a carrier that will guide their path for localized delivery. For example, studies showed a sustained release of recombinant human granulocyte colony-stimulating factor (G-CSF) from chitosan nanoparticles, which were incorporated in poly(e-caprolactone) fibers (Figure 9) [134]. Results demonstrated a faster wound healing rate on nanofiber/nanoparticle groups using rat models. Hence, the incorporation of nanoparticles in electrospun fibers is a potential solution to optimize the therapeutic effects of nanoparticles.

The advantages of using nanoparticles in drug delivery include the ability to enhance solubility of highly hydrophobic drugs, to provide sustained and controlled release of encapsulated drugs, to increase stability of therapeutic agents, to deliver drugs in high concentration, and to provide specific treatment when modified with cell-specific ligands [135]. The most commonly used nanoparticles for biomedical application include polymeric nanoparticles, nano-emulsion, lipid-based nanoparticles, metal nanoparticles and dendrimers. Further studies have shown that composites of nanoparticle-fiber composites have shown improved antibacterial activities. In addition, studies showed that a hybrid composite scaffold, composed of poly(lactic-co-glycolic) acid nanoparticles embedded in chitosan-poly(ethylene oxide) fibers,supported wound healing process by releasing vascular endothelial growth factor and platelet derived growth factor-BB. Results showed a sustained release of platelet derived growth factor-BB from poly(lactic-co-glycolic) acid nanoparticles in fibrous meshes, with 40% of release after 7 days, as compared to 100% release observed in non-composite nanoparticles [136,137]. In another study, gold nanoparticles were embedded in polycaprolactone fibers to form a composite material for differentiation of MSCs in cardiac lineage [138]. Results showed an enhanced proliferation of cardiomyocytes and MSCs using nanoparticle/fiber composites for cell cultures with the ability to stimulate the differentiation of MSCs into cardiogenesis.

## CONCLUSIONS AND FUTURE DIRECTIONS

Stem cell therapy has become a promising method in skin tissue engineering. Traditional stem cell therapy involves in the injection of the cells around the wound site. However, low levels of cell proliferation/ differentiation and high levels of cell death become a challenge due to the microenvironment cues of the surrounding tissue. To better control the viability of the stem cells as well as their phenotypic expressions, the use of biomaterial scaffolds as cell carriers and/or culture substrates appears to be an exciting opportunity in skin tissue regeneration. In this review, we discussed the potential uses of electrospun fibers for stem cell therapy in non-healing and/or chronic wounds. Tissue scaffolds made from electrospun fibers have the advantages in cell signaling and guiding cell growth through fiber morphology and/or fiber structure. In addition, fibers can be incorporated with bioactive agents to provide multi-phase treatment modalities. Furthermore, electrospun fibers are able to strategically incorporate stem cells in a layer-by-layer structure to mimic tissue structure. These advantages demonstrate that electrospun fiber scaffolds are ideal for stem cell therapy in wound healing.

Since almost all polymers can be electrospun into fibers, the choice of the fiber matrix becomes an important topic. For example, fabrications of electrospun fibers from water insoluble polymers typically involve in the use of organic solvents. The residual solvent in the fibers, if not evaporated completely, can introduce cell death during culture. Furthermore, water insoluble polymers typically have a longer degradation time than the water-soluble polymers. As such, these fibers are ideal to serve as cell carriers and provide a controlled release of the bioactive agents. Water-soluble polymers, on the other hand, disintegrate in seconds, if not minutes, after in contact with culture media and/or body fluid. This behavior may not provide any advantage in cell therapy. As such, one of the future directions is to research the feasibility of using blend fibers (exhibiting both properties) in stem cell culture and engineering the effects of fiber degradation on cell viability and cell properties.

Several cell-based products for skin substitutes are available on the market, such as Epicel^™^, Dermagraft®, and Apligraf®. These products generally involved in the use of a substrate for cell culture before implantation of the allogeneic skin dressing back to the patient. While electrospun fibers are compatible in this regard, the mechanical properties of the fiber scaffolds are a major topic for future research. Current research works on fiber mechanical properties are often considered at the as-fabricated fiber mat conditions under dry/ conditioned state. However, the information on mechanical properties would be misleading when fibers are used as a culture substrate or used as a dressing. Fiber degradation/disintegration may also affect the mechanical properties of the fibers. As such, the future direction of using electrospun fibers for stem cell therapy is to have a better understanding of the mechanical properties of the fiber scaffolds.

Stem cell therapy in tissue regeneration remains a hot topic in wound healing with benefits to promote patient health and to reduce the burden in economic and healthcare system. Our review provides extensive discussion on the use of electrospun fibers as tissue scaffolds for stem cell culture and delivery.

## Figures and Tables

**Table 1.** Implications of stem cells in wound healing.

Stem cells	Animal model	Groups	Wound healing	Re-epithelialization	Ref.

ASCs	Yorkshire	High (ASCs) 3.0×10^6^ cells/cm^2^	48% (10 days)	3.7 mm thickness	[51]
	pigs		65% (14 days)	(1 week)	
		Medium (ASC) 1.0 × 10^6^ cells/cm^2^	–	–	
		Low (ASC) 0.3 × 10^6^ cells/cm^2^	–	–	
		Saline injected control	37.9% (10 days)	2.2 mm thickness	
			48.9% (14 days)	(1 week)	
		Untreated negative control	–	–	

ASCs	Mice	(ASCs+PBAE+VEGF)	Prolonged cell survival (day 6 to 10).	Mature collagen fiber	[52]
		PBAE: poly (β-amino) esters	Dermal scar shows mature/round	(14 days)	
			collagen fibers		
		(ASCs)	Dermal scar shows mature/round	Increase cellularity &	
			collagen fibers	collagen (14 days)	
		(PBS)	Dermal scar shows thin/flat	Immature collagen	
			collagen fibers	fiber (14 days)	

AD-MSCs	Mice	Treated diabetic models with	Wound closure increase	(48.5 ± 6.3)% volume	[53]
		(AD-MSCs)		density of collagen	
		Untreated diabetic models were	–	(46.6 ± 7.4)% volume	
		injected with phosphate buffer		density of collagen	
		saline (PBS)-Control group			
		Non-diabetic models (non-DMs) were	–	(42.6 ± 4.4)% volume	
		injected with (PBS)		density of collagen	

BMSCs	Mice	Hydrogel-BMSCs	Remarkable wound healing (day 7)	Promote wound	[54]
				closured	
		BMSCs-alone	–	–	
		Hydrogen-alone	–	–	
		Control group	–	–	

ASCs	Swine	(10 million) ASCs	Wound closure (28 days)	100 ± 0 (28 days)	[55]
		(5 million) ASCs	Wound closure (28 days)	99 ± 0.3 (28 days)	
EC/ASCs		(10 million) EC/ASCs	Wound closure (28 days)	98.1 ± 2.1 (28 days)	
		(5 million) EC/ASCs	Wound closure (28 days)	99.7 ± 0.5 (28 days)	
HUVEC		ASC-CM	Wound closure (28 days)	96.0 ± 3.9 (28 days)	
		HUVEC-CM (human umbilical	Wound closure (28 days)	96.0 ± 3.9 (28 days)	
		endothelial cell)	Wound closure (28 days)		
		Control (Serum-free medium-2 mL)	–	–	

**Table 2.** The use of electrospun in combination with stem cells on wound healing.

Stem cells	Nanofibers	Animal model	Group	Wound healing	Re-epithelialization	Ref.

–	PCL/collagen	Mice	(L-b-L assembled-fibroblasts	31 ± 7% (21 days)	40% (10 days)	[72]
			and keratinocytes)			
			(L-b-L	45 ± 11% (21 days)	20 ± 3% (10 days)	
			assembled-fibroblasts)			
			Acellular nanofiber meshes	Lowest integration (21 days)	11 ± 5% (10 days)	
					21 ± 6% (21 days)	
			Autografts implanted back	Wound closure (21 days)	Re-epithelialization	
			after 1 min		observed	

BM-MSCs	PLGA/collagen	Rats	Negative control	No wound closured (1 to	17% (day 10)	[73]
				14 days)	Large un-epithelialization	
			NFS only	Wound closured (1 to 14 days)	15% (day 10)	
					Un-epithelialization	
			NFS-MSCs (3 × 10^3^ per NFS)	Wound closured (1 to 14 days)	Low cell density	
			NFS-MSCs (3 × 10^6^ per NFS)	Wound closured (1 to 14 days)	High cell density	

MSCs	Poly-D,L-lactic	Mice	Injury without matrices	–	–	[74]
	acid		PDLLA/Sp without MSCs	Similar wound size compared	Showed a great number of	
	(PDLLA)/Sp *			to all groups with better	cells compare to PDLLA	
				cicatrization	group. Mostly cells from	
					the model mice.	
			PDLLA/Sp MSCs	Similar wound size compared	Great number of cells were	
				to all groups with better	observed compare to	
				cicatrization	PDLLA group.	
			PDLLA without MSCs	Similar wound size compared	The growth of cells was	
				to all groups	observed mostly from the	
					model mice.	
			PDLLA with MSCs	Similar size wound	–	

* Sp = *Spirulina*

**Table 3.** Polymeric nanofibers investigated for skin tissue engineering application.

Polymer	Fabrication method	Cell	Result	Ref.
Collagen	Crosslinking Freeze-dry, and electrospinning collagen fibers	Human dermal fibroblast, epidermal keratinocytes	Reduce wound contraction. Skin substitute for fully thicken wound	[77]
Skin fibroin	Electrospinning 3–5 wt% silk fibroin nanofiber	Keratinocytes and fibroblasts	Ideal for wound dressing and tissue engineering	[78]
Gelatin	Electrospinning 10–16 wt% gelatin type B	Human skin fibroblasts	Potential dermal-epidermal skin substitute.	[79]
Poly(3-hydroxy butyrate-*co*-3- hydroxyvalerate)	Electrospinning and solvent casting 15 wt% PHBV	Human skin fibroblasts	Promote in an increase of collagen and re-epithelialization	[80]
Poly(lactide-*co*-glycolide)	Electrospinning PLGA at 0.2 to 0.27 g/mL	Human keratinocytes	Optimize skin fibroblast attachment and growth	[81]
Poly(ε-caprolactone)- gelatin	Electrospinning 10 wt% PCL and 10 wt% gelatin	Human keratinocytes	Enhanced cell infiltration for accelerated dermal wound healing	[82]

**Table 4.** Incorporation of the growth factors in fibers for stem cell therapy in wound healing.

DDS	Method	Growth factor	Study	Result	Ref.
Gelatin/poly(L-lactic acid)-*co*-poly-(ε-capr olactone) nanofiber	Coaxial electrospinning	EGF	Human fibroblasts Adipose-derived stem cells	Complete closure of wound after 9 days	[126]
Activated platelet rich plasma	Blend electrospinning	FGF, VEGF, EGF	*In vitro*	Rapid cellular infiltration	[128]
PELA(PEG-PLA) Poly(ethyleneoxide- *co*-lactic acid)	Core-sheath nanofibers	bFGF	Tested *in vitro* and *in vivo* in diabetic rats	Sustained release of bFGF resulted in complete epithelialization after 4 weeks	[125]
